# Multi-Toxin Occurrences in Ten French Water Resource Reservoirs

**DOI:** 10.3390/toxins10070283

**Published:** 2018-07-09

**Authors:** Frederic Pitois, Jutta Fastner, Christelle Pagotto, Magali Dechesne

**Affiliations:** 1 Limnologie sarl, 16 rue Paul Langevin, 35200 Rennes, France; 2 German Federal Environment Agency (UBA), Corrensplatz 1, 14195 Berlin, Germany; jutta.fastner@uba.de; 3 Veolia Water, 30 rue Madeleine Vionnet, 93300 Aubervilliers, France; christelle.pagotto@veolia.com; 4 Veolia Recherche & Innovation, Chemin de la Digue, 78603 Maisons-Laffitte, France; magali.dechesne@veolia.com

**Keywords:** cylindrospermopsin, anatoxin-a, PSP toxins, microcystins, cyanobacteria, Nostocales, drinking water

## Abstract

Cyanobacteria are known to produce a wide array of metabolites, including various classes of toxins. Among these, hepatotoxins (Microcystins), neurotoxins (Anatoxin-A and PSP toxins) or cytotoxins (Cylindrospermopsins) have been subjected to numerous, individual studies during the past twenty years. Reports of toxins co-occurrences, however, remain scarce in the literature. The present work is an inventory of cyanobacteria with a particular focus on Nostocales and their associated toxin classes from 2007 to 2010 in ten lakes used for drinking water production in France. The results show that potential multiple toxin producing species are commonly encountered in cyanobacteria populations. Individual toxin classes were detected in 75% of all samples. Toxin co-occurrences appeared in 40% of samples as two- or three-toxin combinations (with 35% for the microcystins–anatoxin combination), whereas four-toxin class combinations only appeared in 1% of samples. Toxin co-occurrences could be partially correlated to species composition and water temperature. Peak concentrations however could never be observed simultaneously and followed distinct, asymmetrical distribution patterns. As observations are the key for preventive management and risk assessment, these results indicate that water monitoring should search for all four toxin classes simultaneously instead of focusing on the most frequent toxins, i.e., microcystins.

## 1. Introduction

Cyanobacteria proliferations are a worldwide consequence of lake eutrophication, with potential public health issues for water management, water production and recreational use such as bathing. Despite constant research efforts for the last 30 years, toxin production and occurrence, i.e., why, when and which species will produce any toxin, alone or in any combination with other toxins, is still insufficiently understood. Cyanobacterial toxins include a wide variety of molecules, such as hepatotoxins (microcystins and nodularins), cytotoxins (cylindrospermopsins), neurotoxins such as anatoxin-a, PSP toxins (Paralytic Shellfish Poisoning) or dermatotoxins such as Lyngbyatoxin, a potent dermatitis agent [1].

According to the World Health Organization handbook [2], microcystins (MCs) are the most studied and monitored toxins. MCs are cyclic heptapeptids comprising more than 200 variants [3], have already been reported from most countries, and are known to be produced by many common taxa in continental waters such as *Microcystis*, *Planktothrix*, *Anabaena*/*Dolichospermum*, etc.

Cylindrospermopsin (CYN) is a hepatotoxic alkaloid first identified in an Australian *Cylindrospermopsis raciborskii* [4], then in tropical or subtropical waters [5,6,7,8,9], and recently also in European lakes in Germany, Italy or France [10,11,12]. CYN and congeners have been reported to be produced by species such as *Aphanizomenon flos-aquae*, *A. ovalisporum* [13,14], *Raphidiopsis curvata* and *R. mediterranea* [15].

Anatoxin-A (ATX) is a neurotoxic alkaloid observed worldwide and associated with many common taxa: *Dolichospermum* (from *Anabaena*) *flos-aquae*, *Aphanizomenon flos-aquae* [16], *Dolichospermum planctonicum* [17], *Cuspidothrix* (from *Aphanizomenon*) *issatchenkoï* [18], *Raphidiopsis mediterranea* [19], and *Microcystis aeruginosa* [20].

PSP toxins are neurotoxic alkaloids produced by freshwater cyanobacteria and marine dinoflagellates with more than 30 identified variants such as saxitoxin, neosaxitoxin, decarbamoylsaxitoxin, gonyautoxins, etc. [21]. Known potential PSP producing species include *Cuspidothrix issatchenkoï* [22], *Cylindrospermopsis raciborskii* and *Raphidiopsis brookii* [23,24], *Aphanizomenon flos-aquae* and *A. gracile* [25,26,27], *Dolichospermum circinalis* [1], *Dolichospermum lemmermannii* [28], or *Microcystis aeruginosa* [29].

The dermatotoxins lyngbyatoxin and aplysiatoxin are contact dermatitis and tumor promoting agents produced by benthic species such as *Lyngbya wollei* or *Lyngbya majuscula* [30,31]. These alkaloids have mainly been reported from marine lagoons and subtropical lakes and are under-documented in other contexts, as benthic species are seldom observed in planktic flora surveys.

Besides microcystins, the other toxin classes are less commonly studied and monitored, and data about their simultaneous occurrence are scarce in the literature, with the exception of studies from Italy [32], Germany [33] or the USA [34,35]. ATX, CYN or PSP toxins can thus be considered as “emerging toxins” either because of a recent spread in resource waters, or because of a recent interest for water managers. These toxin classes are mainly produced by taxa from the order Nostocales, i.e., *Dolichospermum*, *Aphanizomenon*, *Cuspidothrix*, *Cylindrospermopsis*, *Raphidiopsis*, etc.

In continental Europe, Nostocales species composition associates autochthonous taxa (*Dolichospermum flos-aquae*, *Aphanizomenon flos-aquae*, and *Aphanizomenon gracile*) with new, invasive species such as *Cylindrospermopsis raciborskii*, *Anabaena bergii* or *Sphaerospermopsis aphanizomenoides* [36]. Various factors have been proposed to explain these invasive species extension, such as transport by migrating birds [37], conjugated with climate change, namely the increase of spring temperature and solar radiation fluxes [38,39,40,41]. Although these invasive species do not appear to be the main CYN producers, dedicated studies have shown CYN to become as frequently detected as MCs in German lakes in the recent years [10,36].

In the French regulatory context, MCs are routinely monitored in resource or bathing waters since 2003, whereas CYN, ATX and PSP toxins are only optionally analyzed since 2013. All toxin classes have, however, already been detected individually: ATX [42,43], CYN [12], and PSP [27] are known to occur in French lakes and rivers but large-scale exploration has never been performed. In this context, this work is an inventory of cyanobacteria with a particular focus on Nostocales and associated toxin classes (MCs, ATX, CYN and PSP), conducted from 2007 to 2010 in 10 freshwater lakes in France. These lakes are used as resources for drinking water production.

## 2. Results

The results presented below were obtained from 10 reservoirs and their associated pre-dams sampled monthly between June and October in 2007, 2008 and 2010. A total of 192 samples were collected, of which 98% contained cyanobacteria, and 70% at least one toxin class.

### 2.1. Cyanobacteria

**Figure 1:**
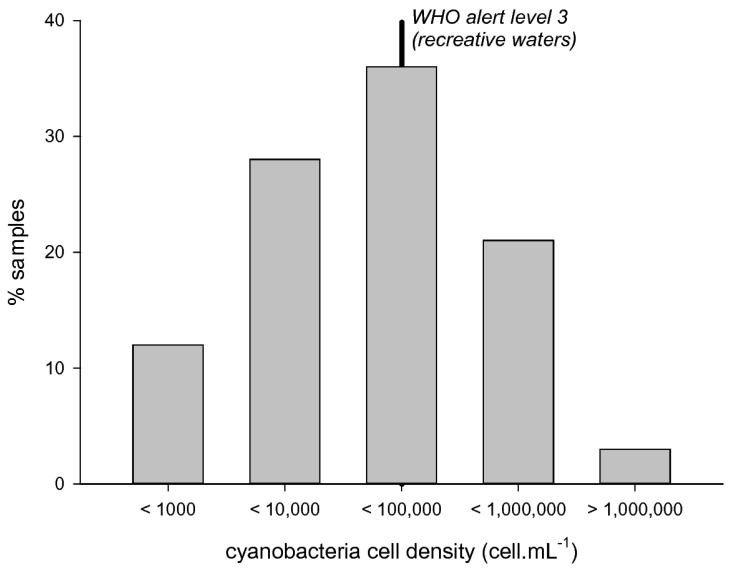
Cyanobacteria cell densities distribution in the samples collected from 2007 to 2010 (*n* = 192).

Cyanobacteria were observed with relatively low cell densities (Figure 1): 12% of samples were below 1000 cell/mL, whereas 41% of samples were higher than 20,000 cell/mL, i.e., WHO alert level 2, and 24% were higher than 100,000 cell/mL, i.e., WHO alert level 3. The highest peak cell density reached 3,320,500 cell/mL in 2007.

The order Chroococcales was the most common order every year, and appeared in 88% of all samples with 18 different taxa. Species from the genera *Aphanothece*, *Snowella*, *Microcystis* (*M. aeruginosa*) or *Worochininia* (*W. compacta*) could be observed in 33–45% of samples. The highest Chroococcales biomass, i.e., 234 mm^3^/L, was attributed to a sample dominated by *Microcystis viridis* reaching 1,308,200 cell/mL in 2010 in lake No. 7.

Oscillatoriales, observed in 85% of samples, were the second most frequent order. Species composition was dominated by *Planktothrix agardhii*, in 62% of samples, and *Phormidium splendidum*, in 37% of samples; 10 other taxa were recorded with low cell densities in less than 14% of samples. The maximal Oscillatoriales biomass, 63 mm^3^/L, was observed in a sample dominated by *P. agardhii* with 1,550,500 cell/mL in 2010 in lake No. 8.

The order Nostocales was observed in 65% of samples with 17 taxa. The most common species were *Cuspidothrix issatschenkoï* and *Aphanizomenon flos-aquae* in 45–47% of samples. Immature Dolichospermum, i.e., without heterocysts and akinetes, were present in 32% of samples, and all other taxa appeared in less than 12% occasions. The peak Nostocales biomass, 504 mm^3^/L, was recorded in a sample dominated by *Dolichospermum flos-aquae* with 3,320,500 cell/mL in 2007 in lake No. 9.

**Figure 2:**
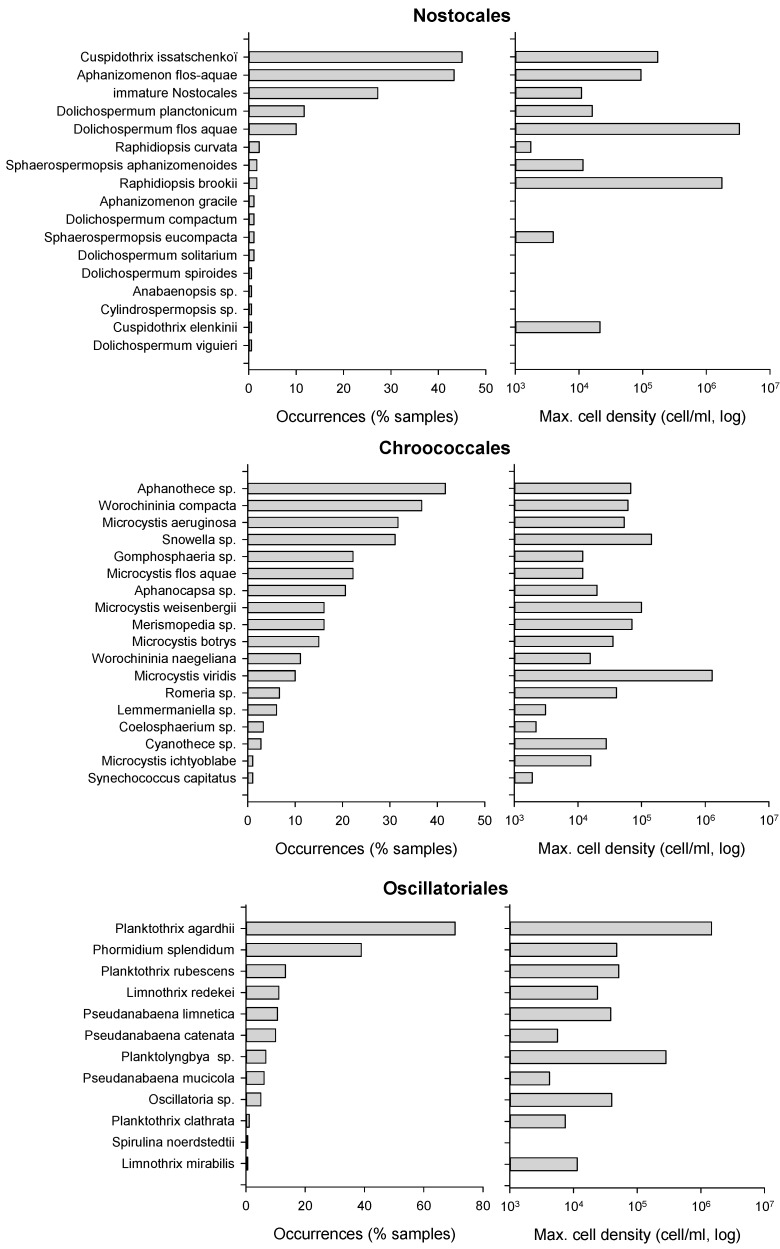
Cyanobacteria taxonomic distribution expressed as occurrence frequency and maximum cell density (*n* = 185).

Cyanobacteria distribution is summarized in Figure 2. Most taxa associated with the highest frequencies or cell densities were common species in the French context, such as *Planktothrix agardhii* and *P. rubescens*, *Aphanizomenon flos-aquae*, *Cuspidothrix issatchenkoï*, *Microcystis aeruginosa*, *M. flos-aquae*, *M. viridis*, etc. Some less common species could however be observed, i.e., Nostocales such as *Dolichospermum compactum* and *D. viguieri*, *Sphaerospermopsis eucompacta*, *Anabaenopsis arnoldii*, *Cuspidothrix elenkinii* and *Aphanizomenon schindleri*, in less than 5% of samples and with cell densities lower than 1000 cell/mL. Only one uncommon species, *Raphidiopsis brookii*, could be observed with a significant biomass of 1,824,000 cell/mL and 156 mm^3^/L, in August and September 2010 in lake No. 6.

From 2007 to 2010, 25 known toxin producing species were recorded, with 18 known MCs producers, 9 known ATX producers, 6 known PSP producers and 4 known CYN producers. Some commons species appear to be potential producers for 2–3 toxin classes. The complete known toxin producing species listing is provided in Table S1.

Compared to analyzed species composition, potential MCs and ATX producers occurred in 92% and 68% of all samples, vs. 65% and 42%, respectively, for known PSP and CYN producers. In the same time, samples containing no cyanobacteria or no known toxin-producing species accounted for, respectively, 3% and 7% of samples.

Samples hosting one toxin-producing species only accounted for 24% of samples, whereas 60% of samples contained species potentially associated with all four toxin classes. This is explained by the widespread distribution of *Aphanizomenon* sp. and *Microcystis aeruginosa*, suspected to be multiple toxin classes producers. These two species were identified in 37% and 29% of samples while occurring concomitantly with other toxin-producing species.

### 2.2. Toxin Classes

First it should be noted that no extracellular toxin could be detected in any analyzed sample, and all exposed results relate to intracellular toxin concentrations.

#### 2.2.1. Microcystins

Microcystins (MCs) were detected in 64% of all analyzed samples, with high interannual variability: 45% of samples were positive in 2007 vs. 20% in 2008 and 100% in 2010. This is corroborated by another study dedicated to 26 lakes in western France, including two lakes and three monitoring years in common with the present study [44], showing that 2007 and 2008, similar to 2004 and 2011, had distinctly lower MCs detection frequencies compared to 2006, 2009 or 2010 where detection frequencies were the highest. Maximal detection frequencies were recorded from 20,000 to 100,000 cell/mL (i.e., between WHO alert thresholds 1 and 2) with 83% MCs detections, whereas maximal concentrations were observed above 100,000 cell/mL with 12.5 µg/L (Table 1).

**Figure 3:**
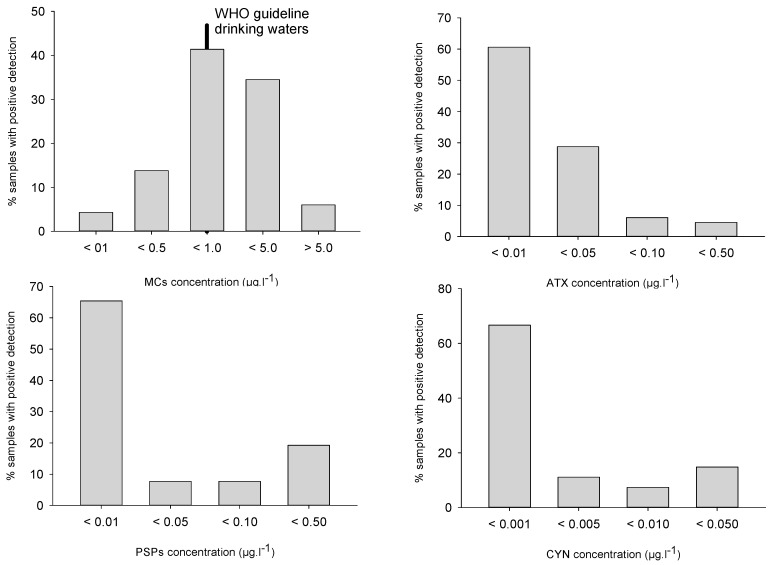
Toxin classes distribution in all analyzed samples (all toxin congeners summed up).

Despite this variability, these frequencies are in broad agreement with already published data, i.e., MCs positive detections in 62–91% of samples analyzed in Italy [32], Germany [33] or the USA [34,35]. MCs concentrations, although substantially higher than the other toxin classes, were mostly lower than 1 µg/L for 60% of samples, and higher than 5 µg/L in 6% of samples (Figure 3). The median (0.55 µg/L) and maximal (12.5 µg/L) values appeared distinctively lower than in other studies, especially compared to Germany [33] and the USA [34,35].

The 2007 and 2008 samples were analyzed for eight MC variants: [Asp3]MC-RR and -LR, and MC-RR, YR, LR, LA, LW and LF. MC-RR and [Asp3]MC-RR, or MC-LR and [Asp3]MC-LR were detected in 94% of samples with mean concentrations of 0.4 ± 1.1 and 0.9 ± 1.3 µg/L respectively. MC-LF was detected in 77% of samples (mean: 0.01 ± 0.02 µg/L), and MC-YR in 71% of samples (mean: 0.05 ± 0.09 µg/L). MC-LW and MC-LA were detected in 63% and 45% of samples respectively, with concentrations lower than 0.03 µg/L.

#### 2.2.2. Anatoxin-A

Anatoxin-A (ATX) was detected in 35% of all samples which is similar to or higher than in USA studies [33,35] (7%) but lower than in German studies (57%) [33]. Once again, interannual variations were observed: 22% of samples were positive in 2007 and 2008, and 50% in 2010. Most samples (88%) showed ATX concentrations lower than 0.05 µg/L whereas concentrations higher than 0.1 µg/L could be observed in 1% of samples (Figure 3). Detection frequency tended to increase with cell density, and reached 60% above 100,000 cell/mL (Table 1). Maximal concentration (0.46 µg/L) appeared distinctly lower than median values reported from Germany [33] and the USA [34]

#### 2.2.3. Cylindrospermopsins

Cylindrospermopsins (CYN) were detected in 15% of samples, i.e., 9–11% of samples in 2007–2008 and 19% of samples in 2010, similar to occurrences in the USA [34] and significantly lower than reported from German lakes (83%) [33]. CYN concentrations were always lower than 0.03 µg/L, with 78% of samples lower than 0.01 µg/L (Figure 3). Deoxy-CYN was also investigated but could not be detected in our samples. Maximal detection frequency (17% of samples) was recorded from 20,000 to 100,000 cell/mL, whereas maximal concentrations were observed in samples with cell densities lower than 20,000 cell/mL (Table 1).

It must be noted that dissolved CYN could not be observed in any sample. This is distinctly different from already reported observations where high extra-cellular concentrations tend to be the main fraction of total CYN (see [45] and references therein for example).

#### 2.2.4. PSP Toxins

PSP were detected in 14% of samples, with 23% of samples in 2007, 11% of samples in 2008 and 10% of samples in 2010 (Figure 3). Once again, this is similar to reported frequencies in the USA [34] and lower than observations from German lakes (69%) [33]. PSP concentrations were mostly (in 73% of samples) lower than 0.005 µg/L, whereas 19% of samples ranged from 0.01 to 0.05 µg/L. Detection frequencies were maximal (i.e., 35% of samples) for cell densities higher than 100,000 cell/mL, whereas similar maximal concentrations could be observed in every cell density class (Table 1). The observed PSP congeners were Saxitoxin (STX) in eight samples, ranging from 0.04 to 0.15 µg/L, and Gonyautoxin-5 (GTX-5) in four samples, ranging from 0.03 to 0.08 µg/L. Other congeners were not detected in any sample.

### 2.3. Toxin Classes Distribution

Multiple toxin combinations appeared in 40% of all samples, mostly as a two-toxin class combination (27% of samples), whereas three toxins could be detected in 12% of samples and four toxin classes in 1% of samples. At the same time, 35% of samples hosted only one toxin class, mainly MCs. This appears in close agreement with observations in Midwestern USA lakes [34] where multiple toxin combinations were observed in 48% of samples, with two toxin classes in 30% of samples and three toxin classes in 18% of samples.

**Figure 4:**
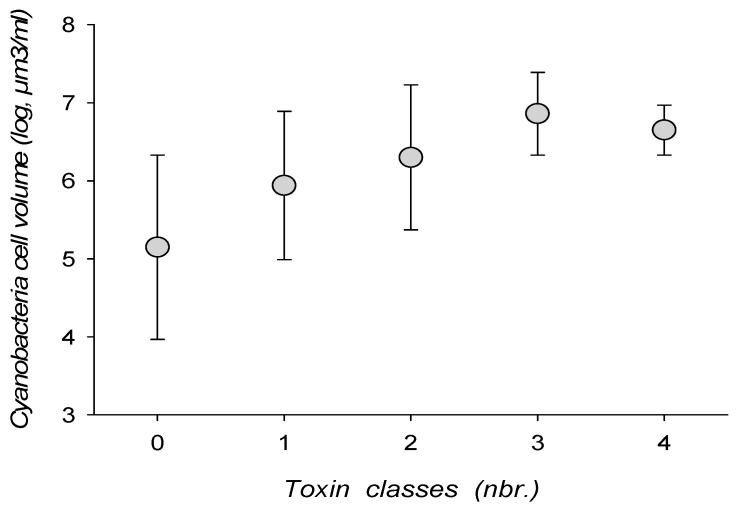
Toxin class associations and cyanobacteria total cell biovolume.

Toxin combinations tended to increase with cyanobacterial biomass, either expressed as cell density or cell biovolume (Figure 4). Combinations of 3–4 toxin classes could, however, be encountered with a cell density as low as 7605 cell/mL, or a cell volume of 0.59 mm^3^/mL.

**Figure 5:**
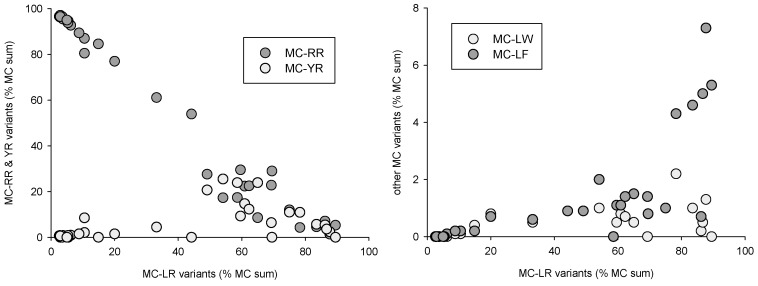
Microcystin variant combinations expressed as percent of total measured MCs.

Distinct distribution patterns could be observed for the various toxin classes and toxin variants. Microcystin variants, when expressed as percent of total MCs (Figure 5), could be separated into two groups, with either -RR/-[Asp3]RR or -LR/[Asp3]LR dominated samples. In the -LR-dominated group, MC-YR, LW and LF tended to first increase simultaneously to MC-LR, and then decrease when -LR reached 60% of total MCs. This indicates that MC-positive samples were composed either of (mostly) -RR and -[Asp3]RR variants, or of (mostly) -LR and [Asp3]LR variants associated with low concentrations of -YR, LW and LF microcystins. Only one of all samples had a nearly equal composition with 54% MC-RR vs. 46% MC-LR.

Similar MCs variant distribution patterns can be observed in the results from the USA [34]. In our case, this distribution can be partly attributed to cyanobacteria species composition: MC-RR was correlated with *Planktothrix* and *Aphanizomenon* biomass (*r*² = 0.47 and 0.43, respectively, *p* < 0.01), MC-YR and MC-LF correlated with *Microcystis* biomass (*r*² = 0.38 and 0.34, respectively, *p* < 0.01), and [Asp3]MC-LR with *Dolichospermum* biomass (*r*² = 0.36, *p* < 0.01). MC-LR, on the other hand, could not be correlated with any species group, which is consistent with a possible production by nearly all potentially toxic species observed in the samples. It can thus be hypothesized that MCs variant distribution is a direct consequence of species successional patterns.

**Figure 6:**
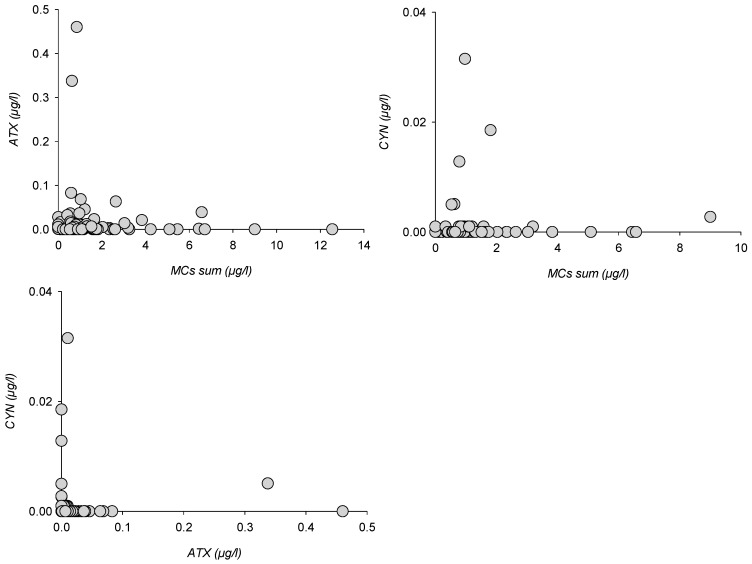
Paired toxin classes distribution for MCs, ATX and CYN.

Other toxin classes could not be analyzed for enough variants or observed in enough samples to show species-controlled distributions. Paired toxin distribution could, however, be compared for MCs vs. ATX (123 samples), MCs vs. CYN (*n* = 122) and ATX vs. CYN (*n* = 62), whereas quantified PSP were insufficiently numerous to allow for a comparison (Figure 6).

ATX and CYN peaks appeared asymmetrically distributed when compared to total MCs, as peak concentrations were recorded for lower MCs, whereas MCs peaks corresponded with lower ATX or CYN concentrations. Similarly, ATX and CYN concentrations were conversely distributed relatively to each other. Whichever class is considered, no sample hosted simultaneous peaks with two or more toxins. When compared with MCs distribution patterns, this means that any peak sample was composed of either [Asp3]MC-RR and MC-RR or [Asp3]MC-LR and MC-LR dominated MCs, or ATX, or CYN.

### 2.4. Measured Environmental Parameters

Statistical analyses were unable to show direct relations among environmental parameters, cyanobacteria and toxins. However, some indirect, non-significant relations appeared: Cyanobacteria cell densities or biomasses were higher in unstratified (vs. stratified) or shallower lakes with higher turbidity (i.e., lower Secchi depths), and tended to increase with water temperatures. Microcystin concentrations increased with higher cyanobacterial biomass. Toxin classes number increased with mean water temperatures (in unstratified lakes) or with mean temperatures in the euphotic layer (in stratified lakes), along with cyanobacteria cell densities or biomass.

These results could be expected as previous studies have shown that individual toxin classes production could be related to abiotic parameters such as temperature or light, whereas shallow, unstratified lakes are known to show higher primary production and higher planktonic biomass than deeper, stratified lakes. However, if we assume that toxin concentration in a lake depends on: (1) the biomass of all potentially toxin-producing species; (2) their genotype composition; and (3) the type and amount of toxin each genotype produces, environmental factors can act on all levels. It is unclear then whether the observed toxin distribution is a direct consequence of water temperature, i.e., higher temperatures leading to higher toxin production, or an indirect consequence of site characteristics on cyanobacteria populations, i.e., temperature and planktonic diversity leading to higher probability for any lake to host one or more species producing one or more toxin classes.

## 3. Conclusions

Regarding cyanobacteria, expected species such as invasive, allochthonous Nostocales remained rarely encountered during the three sampling years. Some uncommon taxa from the genera *Cylindrospermopsis*, *Anabaenopsis*, *Anabaena* or *Aphanizomenon* were observed but remained lower than 1000 cell/mL. With the exception of a *Raphidiopsis brookii* bloom reaching nearly 2,000,000 cell/mL in 2010, all proliferation episodes were related to common taxa in the French context. The relations between water temperatures, lake depth and water stratification indicate that exposure to high cyanobacterial biomass and multiple toxin classes occurring simultaneously are more likely in shallower, unstratified lakes such as smaller artificial lakes devoted to bathing and other recreative activities. This can also be the case in pre-dam lakes, where waters are often eutrophic because of the nutrient load provided by the lake tributaries, whereas temperatures were often 1–2 °C higher than in the main reservoir. In this sense, pre-dams can act as incubators contaminating the main lakes with dense cyanobacteria inoculum.

Regarding toxin classes, microcystins, as expected, were the most common with 64% positive samples and concentrations similar to already observed values in France and ranging from 1 to 10 µg/L. Anatoxin-A was the second most frequent toxin class with 34% positive samples whereas maximum concentration was inferior to 0.5 µg/L. PSP toxins and cylindrospermopsin, on the other hand, appeared fairly uncommon with 14–15% positive samples and maximum concentrations lower than 0.15 and 0.05 µg/L respectively. Toxin concentrations and toxin class frequencies appeared positively related to cyanobacterial biomass and water temperatures. In this sense, the fairly low multiple toxin occurrence frequencies observed in 2007 and 2008 compared to 2010 could be explained by the relatively unstable meteorological conditions encountered during these summers, as for most sites 2007 and 2008 were the coldest summers since 1993.

The results also show that analyzing MC-LR as an indicator of MCs occurrence or total MCs concentration is inadequate, as MCs variants do not appear evenly distributed in samples, with [Asp3]MC-LR/MC-LR, YR, LA, and LF anticorrelated with [Asp3]MC-RR/MC-RR. Similarly, considering total MCs concentration as indicative of other toxin occurrences is questionable, as peak concentrations for the analyzed toxins were also anticorrelated and simultaneous peaks were never observed.

All our analyses were conducted on concentrated samples and allowed to detect low toxin concentrations. Most present-day toxin monitoring however relies on higher quantification limits, typically 0.2 µg/L for any toxin class in France. If these quantification limits were applied to our results, MCs would have been detected in 59% of samples vs. 64%, ATX in 1% of samples vs. 34%, and PSP or CYN would not have been observed. Regarding toxin detections, in the French context, toxin analysis tends to be concentrated on samples with cell densities higher than WHO alert level 2 (i.e., 100,000 cell/mL). Our results however show that MCs and ATX may appear with significant frequencies and concentrations even in samples with low cell densities, indicating that toxin monitoring should be extended to WHO alert level 1 (i.e., 20,000 cell/mL).

Finally, although invasive cyanobacteria did not appear, all investigated toxin classes could be observed with significant frequencies and low concentrations which then did not represent an acute risk for drinking water production. However, this indicates that survey efforts should not only be directed toward acute toxin concentrations, but should also encompass the consequences of chronic exposure to low cyanobacterial biomass or to cyanobacterial aerosols, such as allergenicity [46,47,48], or to subacute toxin concentrations, such as cytotoxicity [49,50,51].

## 4. Materials and Methods

### 4.1. Sites

**Figure 7:**
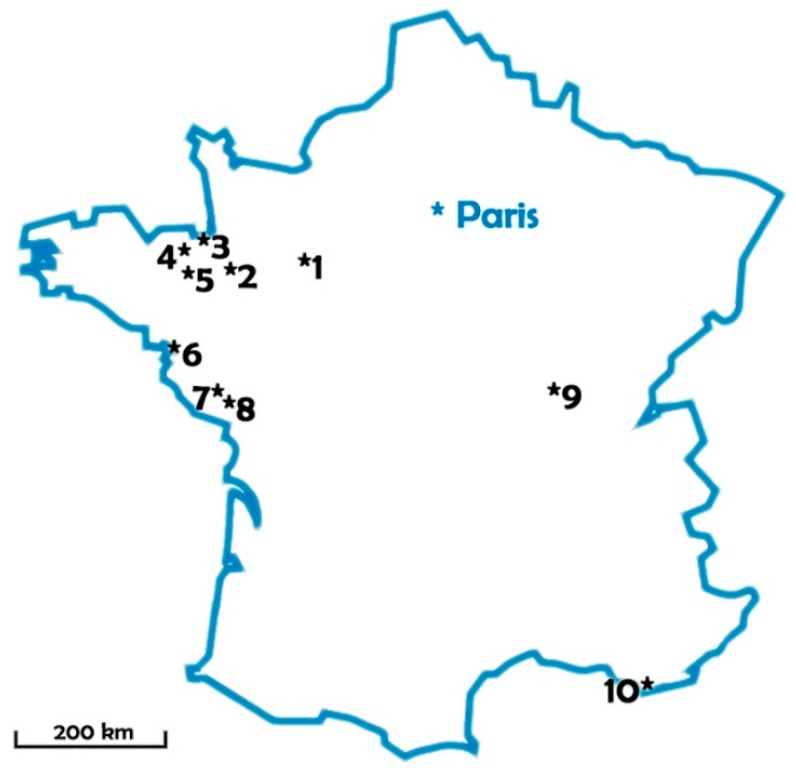
Sampled lakes location.

Sampling campaigns were conducted during the summers 2007, 2008 and 2010 in 10 lakes used as freshwater resources for drinking water production: eight in western France under oceanic climate, one in center-east under semi-continental climate, and one in the south under Mediterranean climate. Lake localization and volumes are summarized in Figure 7 and Table 2.

### 4.2. Sampling and Sample Processing

During these three years, 192 integrated water samples were collected. All samples were collected monthly from June to October, as French institutional monitoring data show that cyanobacterial proliferations have the highest probability to occur during these months. Secchi depth was measured with a Secchi disk, and samples were collected in the deepest part of the lakes and of their pre-dams with a Van Dorn sampling bottle and integrated within the euphotic depth. On every occasion, vertical profiles for pH, dissolved oxygen, conductivity and temperature were recorded every 50 cm with an YSI 556 MPS multiparameter probe.

On the sampling day, all samples were separated into two batches: a batch for plankton analysis was fixed with alkaline Lugol solution, whereas a batch for toxin analysis was filtered on Sartorius regenerated cellulose (0.45 µm). Filters and filtered water were then frozen separately until further analysis.

#### 4.2.1. Species Composition Analysis

All samples were analyzed for species distribution by Limnologie sarl on the day following sampling under a Leica DMLS light microscope with phase contrast using common reference floras [52,53,54,55,56,57,58,59,60]. Results were expressed for all taxa as cell density (cell/mL) and cell biovolume (mm^3^/L) calculated from cell density and mean cell dimensions.

#### 4.2.2. Toxin Analysis

Filters were extracted three times with acetonitrile–water–formic acid (80:19.9:0.1), as previously described by Dell’Aversano et al. [61], and the combined supernatants were dried. Frozen filtrates (2 mL) were thawed and acidified with formic acid to a final volume of 0.1% formic acid. Filtrates were then dried by vacuum centrifugation and stored frozen at −20 °C. Prior to analysis extracts were re-dissolved in 500 µL 75% aqueous acetonitrile for analyses of PSPs, 50% aqueous methanol for microcystin analysis, while for analyses of CYN, D-CYN and ATX aliquots were re-dissolved in 0.1% formic acid. In some cases, samples were further concentrated for unequivocal toxin identification.

##### Analysis by LC-MS/MS: MCs

The extracts were separated using a Purospher STAR RP-18 end-capped column (30 × 4 mm, 3 μm particle size, Merck, Darmstadt, Germany) at 30 °C as described by Spoof et al. [62]. The mobile phase consisted of 0.5% formic acid (A) and acetonitrile with 0.5% formic acid (B) at a flow rate of 0.5 mL/min with the following gradient program: 0 min 25% B, 10 min 70% B, 11 min 70% B. The injection volume was 10 μL. Identification and quantification of the MCs ([Asp3]-MC-RR, MC-RR, MC-YR, [Asp3]-MC-LR, MC-LR, MC-LW, MC-LF, MC-LA, standards purchased at Enzo Life Sciences, Lörrach, Germany) was performed in the MRM (Multiple Reaction Monitoring) mode with the transitions given by Fastner et al. [63].

##### Analysis by LC-MS/MS: CYN, deoxyCYN, ATX

Analyses for CYN, deoxyCYN and ATX were carried out on an Agilent 2900 series HPLC system (Agilent Technologies, Waldbronn, Germany) coupled to a API 5500 QTrap mass spectrometer (AB Sciex, Framingham, MA, USA) equipped with a turbo-ionspray interface. The extracts were separated using a 5 µm Waters Atlantis C18 column (2.1–150 mm) at 30 °C. The HPLC was set to deliver a linear gradient from 1% to 25% MeOH in water, both containing 0.1% formic acid, within 5 min at a flow rate of 0.25 mL min−1. The mass spectrometer was operated in the multiple reaction-monitoring mode (MRM). For the determination of CYN deoxyCYN, and ATX the transitions given in [63] were used. Quantitation of CYN, deoxyCYN and ATX was performed with the most intensive transition. Standard curves were established for all toxins (CYN was obtained from National Research Council, Canada, deoxyCYN from Novakits, Nantes, France, and ATX-a from Tocris, Bristol, UK) and analyzed in a line with the unknowns (one calibration curve after 30 unknowns).

##### Analysis by LC-MS/MS: PSPs

Analyses for paralytic shellfish poisons (PSP) were carried out on an Agilent 1100 series HPLC system (Agilent Technologies, Waldbronn, Germany) coupled to a API 4000 triple quadrupole mass spectrometer (AB Sciex, USA) equipped with a turbo-ionspray interface. The extracts were separated using a 5 µm TSK gel Amide-80, 2 × 250 mm column (Tosoh, Stuttgart, Germany) at 30 °C. The mobile phase consisted of water (A) and acetonitrile-water (95:5, B) both containing 2.0 mM ammonium formate and 3.6 mM formic acid (pH 3.5) at a flow rate of 0.2 mL min^−1^. For the analysis of multiple toxins (cylindrospermopsin, anatoxin-a, paralytic shellfish poisons) the following gradient program was applied: 75% B for 5 min, 75–65% B over 1 min, hold for 13 min, 65–45% over 4 min, hold for 10 min. The mass spectrometer was operated in the multiple reaction monitoring mode for the detection and quantification of the following toxins as described by Dell’Aversano et al. [61]: saxitoxin (STX); neosaxitoxin (NEO); decarbamoylsaxitoxin (dcSTX) and decarbamoylneosaxitoxin (dcNEO); gonyautoxin-1, -2, -3, -4, and -5 (GTX-1, -2, -3, -4, and -5); decarbamoylgonyautoxin (dcGTX-3, -3); and *N*-sulfogonyautoxins-1 and -2 (C1 and C2). Standard curves were established for all the toxins (PSP standards were obtained from National Research Council, Canada) and analyzed in a line with the unknowns (one calibration curve after 20 unknowns).

Limits of quantification (LOQ) for the different toxins at injection of 10 µL sample are as follows: microcystins 0.04–0.5 µg/L depending on congener, CYN 0.01 µg/L, deoxyCYN 0.02 µg/L, ATX 0.02 µg/L and Saxitoxins 0.1–2 µg/L depending on variants. As particulate toxins have been concentrated by filtration between 25 and 300 mL of lake water, LOQs for the particulate toxins were lower than those given above and have been verified for each sample individually based on a signal to noise ratio of 10.

All toxin values ranging from method detection limit (MDL) and method quantification limit (MQL) were considered as unquantified positive detections and accounted for in toxin frequency calculations.

#### 4.2.3. Data analysis

All results were statistically analyzed in search of relations among field data, species composition, biomass and toxin concentrations.

Tested variables included: lake maximum depth, Secchi depth, euphotic depth/max depth, surface water temperature, mean euphotic zone temperature, thermal gradients, thermal stratification, air temperature, cumulated rain and solar radiation, cyanobacteria cell densities, and toxin classes concentrations. All data were separated into 3–5 classes and subjected to Kruskal–Wallis ANOVA with XLSTAT v. 2011.1 software (Addinsoft sarl, Paris, France).

## Figures and Tables

**Table 1:** Toxin detection frequencies (Det: percent of samples) and maximal concentrations (Max.: µg/L) vs. cyanobacteria cell density classes expressed as WHO alert thresholds. **MCs:** total Microcystins, ATX: Anatoxin-a, CYN: Cylindrospermopsin, STX: Saxitoxins

Cell Density Classes	MCs		ATX		CYN		STX	
	Det.	Max.	Det.	Max.	Det.	Max.	Det.	Max.
< 20,000 cell/mL	42%	6.7	18%	0.03	8%	0.03	6%	0.05
20 to 100,000 cell/mL	83%	9.0	47%	0.46	17%	0.01	11%	0.05
> 100,000 cell/mL	79%	12.5	60%	0.34	9%	0.02	35%	0.05

**Table 2:** Lake volumes. Pre-dams were included in the monitoring program.

Lake Number	Lake Volume (m^3^)	Pre-Dam
1	5,700,000	+
2	204,000	+
3	1,335,000	
4	5,000,000	
5	14,700,000	
6	1,100,000	
7	4,400,000	+
8	5,800,000	+
9	10,000,000	+
10	8,000,000	

## Supplementary Materials

The following are available online at http://www.mdpi.com/2072-6651/10/7/283/s1, Table S1: Observed cyanobacterial taxa with known or suspected (in parenthesis) potential toxin production.

## References

[1] Sivonen K., Jones G., Chorus I., Bartram J. (1999). Chapter 3: Cyanobacterial toxins. Toxic Cyanobacteria in Water: A Guide to Their Public Health Consequences, Monitoring and Management.

[2] Chorus I., Bartram J. (1999). Toxic Cyanobacteria. Water. A Guide to Their Public Health Consequences, Monitoring and Management.

[3] Spoof L., Catherine A., Meriluoto J., Spoof L., Codd G.A. (2017). Appendix 3: Tables of Microcystins and Nodularins. Handbook of Cyanobacterial Monitoring and Cyanotoxins Analysis.

[4] Ohtani I., Moore R.E., Runnegar M.T.C. (1992). Cylindrospermopsin, a potent hepatotoxin from the blue-green alga *Cylindrospermopsis raciborskii*. J. Am. Chem. Soc..

[5] Burns J., Chapman A., Williams C., Flewelling L., Carmichael W., Pawlowicz M. Cyanotoxic Blooms in Florida’s (USA) Lakes, Rivers and Tidal River Estuaries: The Recent Invasion of Toxigenic *Cylindrospermopsis raciborskii* and Consequences for Florida’s Drinking Water Supplies. Proceedings of the IX Conference on Harmful Algal Blooms.

[6] Li R., Carmichael W., Brittain S., Eaglesham G., Shaw G., Mahakhant A., Noparatnaraporn N., Yongmanitchai W., Kaya K., Watanabe M. (2001). Isolation and identification of the cyanotoxin cylindrospermopsin and deoxy-cylindrospermopsin from a Thailand strain of *Cylindrospermopsis raciborskii* (Cyanobacteria). Toxicon.

[7] Stirling D., Quilliam M.A. (2001). First report of the cyanobacteria toxin cylindrospermopsin in New Zealand. Toxicon.

[8] Chonudomkul D., Yongmanitchai W., Theeragool G., Kawachi M., Kasai F., Kaya K., Watanabe M. (2004). Morphology, genetic diversity, temperature tolerance and toxicity of *Cylindrospermopsis raciborskii* (Nostocales, Cyanobacteria) strains from Thailand and Japan. FEMS Microbiol. Ecol..

[9] Berry J.P., Lind O. (2010). First evidence of “paralytic shellfish toxins” and cylindrospermopsin in a Mexican freshwater system, Lago Catemaco, and apparent bioaccumulation of the toxins in “tegogolo” snails (*Pomacea patula catemacensis*). Toxicon.

[10] Fastner J., Heinze R., Humpage A.R., Mischke U., Eaglesham G.K., Chorus I. (2003). Cylindrospermopsin occurrence in two German lakes and preliminary assessment of toxicity and toxin production of *Cylindrospermopsis raciborskii* (Cyanobacteria) isolates. Toxicon.

[11] Manti G., Mattei D., Messineo V., Melchiorre S., Bogialli S., Sechi N., Casiddu P., Luglie A., Di Brizio M., Bruno M. (2005). First report of *Cylindrospermopsis raciborskii* in Italy. Harmful Algae News.

[12] Brient L., Lengronne M., Bormans M., Fastner J. (2009). First occurrence of cylindrospermopsin in freshwater in France. Environ. Toxicol..

[13] Banker R., Teltsch B., Sukenik A., Carmeli S. (2000). 7-Epicylindrospermopsin, a toxic minor metabolite of the cyanobacterium *Aphanizomenon ovalisporum* from Lake Kinneret, Israel. J. Nat. Prod..

[14] Preussel K., Stüken A., Wiedner C., Chorus I., Fastner J. (2006). First report on cylindrospermopsin producing *Aphanizomenon flos-aquae* (Cyanobacteria) isolated from two German lakes. Toxicon.

[15] McGregor G., Sendall B., Hunt L., Eaglesham G. (2011). Report of the cyanotoxins cylindrospermopsin and deoxy-cylindrospermopsin from *Raphidiopsis mediterranea* Skuja (Cyanobacteria/Nostocales). Harmful Algae.

[16] Rapala J., Sivonen K., Luukkainen R., Niemela S. (1993). Anatoxin-a concentration in Anabaena and Aphanizomenon under different environmental conditions and comparison of growth by toxic and non-toxic Anabaena strains laboratory study. J. Appl. Phycol..

[17] Bruno M., Barbini D., Pierdominici E., Serse A.P., Ioppolo A. (1994). Anatoxin-A and a previously unknown toxin in *Anabaena planctonica* from blooms found in lake Mulargia (Italy). Toxicon.

[18] Wood S.A., Selwood A.I., Rueckert A., Holland P., Milne J.R., Smith K.F., Smits B., Watts L.F., Cary C.S. (2007). First report of homoanatoxin-a and associated dog neurotoxicosis in New Zealand. Toxicon.

[19] Namikoshi M., Murakami T., Watanabe M.F., Oda T., Yamada J., Tsujimura S. (2003). Simultaneous production of homoanatoxin-a, anatoxin-a, and a new nontoxic 4-hydroxyhomoanatoxin-a by the cyanobacterium *Raphidiopsis mediterranea* Skuja. Toxicon.

[20] Park H.D., Watanabe M.F., Harada K.I., Nagai H., Suzuki M., Watanabe M., Hayashi H. (1993). Hepatotoxin (microcystin) and neurotoxin (anatoxin-a) contained in natural blooms and strains of cyanobacteria from Japanese freshwaters. Nat. Toxins.

[21] Aráoz R., Molgó J., Tandeau de Marsac N. (2010). Neurotoxic cyanobacterial toxins. Toxicon.

[22] Nogueira I.C., Pereira P., Dias E., Pflugmacher S., Wiegand C., Franca S., Vasconcelos V.M. (2004). Accumulation of paralytic shellfish toxins (PST) from the cyanobacterium *Aphanizomenon issatschenkoi* by the cladoceran *Daphnia magna*. Toxicon.

[23] Lagos N., Onodera H., Zagatto P.A., Andrinolo D., Azevedo S.M.F.Q., Oshima Y. (1999). The first evidence of paralytic shellfish toxins in the fresh water cyanobacterium *Cylindrospermopsis raciborskii* isolated from Brazil. Toxicon.

[24] Yunes J.S., De La Rocha S., Giroldo D., Da Silveira S.B., Comin R., Bicho M.D.S., Melcher S.S., Santanna C.L., Vieira A.A.H. (2009). Release of carbohydrates and proteins by a subtropical strain of *Raphidiopsis brookii* (Cyanobacteria) able to produce saxitoxin at three nitrate concentrations. J. Phycol..

[25] Pereira P., Li R., Carmichael W., Dias E., Franca S. (2004). Taxonomy and production of paralytic shellfish toxins by the freshwater cyanobacterium *Aphanizomenon gracile*. Eur. J. Phycol..

[26] Ballot A., Fastner J., Wiedner C. (2010). Paralytic shellfish poisoning toxin-producing cyanobacterium *Aphanizomenon gracile* in Northeast Germany. Appl. Environ. Microbiol..

[27] Ledreux A., Thomazeau S., Catherine A., Duval C., Yéprémian C., Marie A., Bernard C. (2010). Evidence for saxitoxins production by the cyanobacterium *Aphanizomenon gracile* in a French recreational water body. Harmful Algae.

[28] Rapala J., Robertson A., Negri A.P., Berg K.A., Tuomi P., Lyra C., Erkomaa K., Lahti K., Hoppu K., Lepistö L. (2005). First Report of Saxitoxin in Finnish Lakes and Possible Associated Effects on Human Health. Environ. Toxicol..

[29] Santanna C., de Carvalho L.R., Fiore M.F., Silva-Stenico M.E., Lorenzi A.S., Rios F.R., Konno K., Lagos N. (2011). Highly Toxic *Microcystis aeruginosa* Strain, Isolated from Sao Paulo—Brazil, Produce Hepatotoxins and Paralytic Shellfish Poison Neurotoxins. Neurotox Res..

[30] Quiblier C., Wood S., Echenique-Subiabre I., Heath M., Villeneuve A., Humbert J.-F. (2013). A review of current knowledge on toxic benthic freshwater cyanobacteria—Ecology, toxin production and risk management. Water Res..

[31] Pearson L.A., Dittmann E., Mazmouza R., Ongley S.A., D’Agostino P.M., Neilan B.A. (2016). The genetics, biosynthesis and regulation of toxic specialized metabolites of cyanobacteria. Harmful Algae.

[32] Messineo V., Bogialli S., Melchiorre S., Sechi N., Lugliè A., Casiddu P., Mariani M.A., Padedda B.M., Di Corcia A., Mazza R. (2009). Cyanobacterial toxins in Italian freshwaters. Limnologica.

[33] Dolman A.M., Rücker J., Pick F., Fastner J., Rohrlack T., Mischke U., Wiedner C. (2012). Cyanobacteria and cyanotoxins: The influence of nitrogen versus phosphorus. PLoS ONE.

[34] Graham J., Loftin K.A., Meyer M.T., Ziegler A.C. (2010). Cyanotoxin mixtures and taste-and-odor compounds in cyanobacterial blooms from the Midwestern United States. Environ. Sci. Technol..

[35] Backer L.C., Manassaram-Baptiste D., LePrell R., Bolton B. (2015). Cyanobacteria and algae blooms: Review of health and environmental data from the Harmful Algal Bloom-Related Illness Surveillance System (HABISS) 2007–2011. Toxins.

[36] Stüken A., Rücker J., Endrulat T., Preußel K., Hemm M., Nixdorf B., Karsten U., Wiedner C. (2006). Distribution of three alien cyanobacterial species (Nostocales) in northeast Germany: *Cylindrospermopsis raciborskii*, *Anabaena bergii* and *Aphanizomenon aphanizomenoides*. Phycologia.

[37] Cellamare M., Leitão M., Coste M., Dutartre A., Haury J. (2010). Tropical phytoplankton taxa in Aquitaine lakes (France). Hydrobiologia.

[38] Gugger M., Molica R., Le Berre B., Dufour P., Bernard C., Humbert J.F. (2005). Genetic diversity of Cylindrospermopsis strains (cyanobacteria) isolated from four continents. Appl. Environ. Microbiol..

[39] Wiedner C., Rucker J., Bruggemann R., Nixdorf B. (2007). Climate change affects timing and size of populations of an invasive cyanobacterium in temperate regions. Oecologia.

[40] Rücker J., Tingwey E.I., Wiedner C., Anu C.M., Nixdorf B. (2009). Impact of the inoculum size on the population of Nostocales cyanobacteria in a temperate lake. J. Plankton Res..

[41] O’Neil J.M., Davis T.W., Burford M.A., Gobler C.J. (2012). The rise of harmful cyanobacteria blooms: The potential roles of eutrophication and climate change. Harmful Algae.

[42] Gugger M., Lenoir S., Berger C., Ledreux A., Druart J.C., Humbert J.F., Guette C., Bernard C. (2005). First report in a river in France of the benthic cyanobacterium *Phormidium favosum* producing anatoxin-a associated with dog neurotoxicosis. Toxicon.

[43] Cadel-Six S., Peyraud-Thomas C., Brient L., Tandeau de Marsac N., Rippka R., Méjean A. (2007). Different genotypes of anatoxin-producing cyanobacteria coexist in the Tarn River, France. Appl. Environ. Microbiol..

[44] Pitois F., Vezie C., Thoraval I., Baurès E. (2016). Improving Microcystin monitoring relevance in recreative waters: A regional case-study (Brittany, Western France, Europe). Int. J. Hygiene Environ. Health.

[45] Rücker J., Stüken A., Nixdorf B., Fastner J., Chorus I., Wiedner C. (2007). Concentrations of particulate and dissolved cylindrospermopsin in 21 Aphanizomenon-dominated temperate lakes. Toxicon.

[46] Bernstein J.A., Ghosh D., Levin L.S., Zheng S., Carmichael W.W., Lummus Z., Bernstein L. (2011). Cyanobacteria: An unrecognized ubiquitous sensitizing allergen?. Allergy Asthma Proc..

[47] Genitsaris S., Kormas K., Moustaka-Gouni M. (2011). Airborne Algae and Cyanobacteria: Occurrence and Related Health Effects. Front. Biosci..

[48] Ohkouchi Y., Tajima S., Nomura M., Itoh S. (2015). Inflammatory responses and potencies of various lipopolysaccharides fromb acteria and cyanobacteria in aquatic environments and water supply systems. Toxicon.

[49] Gacsi M., Antal O., Vasas G., Mathe C., Borbely G., Saker M.L., Gyori J., Farkas A., Vehovszky A., Banfalvi G. (2009). Comparative study of cyanotoxins affecting cytoskeletal and chromatin structures in CHO-K1 cells. Toxicol. In Vitro.

[50] Nováková K., Bláhaa L., Babica P. (2012). Tumor promoting effects of cyanobacterial extracts are potentiated by anthropogenic contaminants—Evidence from in vitro study. Chemosphere.

[51] Kozdeba M., Borowczyk J., Zimolag E., Wasylewski M., Dziga D., Madeja Z., Drukala J. (2014). Microcystin-LR affects properties of human epidermal skin cells crucial for regenerative processes. Toxicon.

[52] Geitler L. (1932). Cyanophyceae von Europa, Kryptogamen Flora von Deutschland, Osterreich und der Schweiz.

[53] Hill H. (1972). A new Raphidiopsis species (Cyanophyta, Rivulariaceae) from Minnesota lakes. Phycologia.

[54] Komárek J., Anagnostidis K. (1998). Cyanoprokaryota—1 Teil: Chroococcales, Süßwasserflora von Mitteleuropa.

[55] Komárek J., Anagnostidis K. (2005). Cyanoprokaryota—2 Teil: Oscillatoriales, Süßwasserflora von Mitteleuropa.

[56] Watanabe M. (1991). Studies on the Planktonic Blue-Green Algae 3. Some Aphanizomenon Species in Hokkaido, Northen Japan. Bull. Nat. Sci. Mus. Tokyo Ser. B.

[57] Watanabe M. (1992). Studies on Planktonic Blue-green Algae 4. Some Anabaena species with straight Trichomes in Japan. Bull. Nat. Sci. Mus. Tokyo Ser. B.

[58] Watanabe M. (1998). Studies on Planktonic Blue-green Algae 8. Anabaena species with twisted Trichomes in Japan. Bull. Nat. Sci. Mus. Tokyo Ser. B.

[59] Watanabe M., Niiyama Y., Tuji A. (2004). Studies on Planktonic Blue-green Algae 10. Classification of Planktonic Anabaena with coiled Trichomes maintained In the National Science Museum. Tokyo. Bull. Nat. Sci. Mus. Tokyo Ser. B.

[60] Komárek J., Komárkova J. (2006). Diversity of Aphanizomenon-like cyanobacteria. Czech Phycol. Olomouc.

[61] Dell’Aversano C., Eaglesham G.K., Quilliam M.A. (2004). Analysis of cyanobacterial toxins by hydrophilic interaction liquid chromatography-mass spectrometry. J. Chromatogr. A.

[62] Spoof L., Vesterkvist P., Lindholm T., Meriluoto J. (2003). Screening for cyanobacterial hepatotoxins, microcystins and nodularin in environmental water samples by reversed-phase liquid chromatography–electrospray ionisation mass spectrometry et al. J. Chromatogr. A.

[63] Fastner J., Beulker C., Geiser B., Hoffmann A., Kröger R., Teske K., Hoppe J., Mundhenk L., Neurath H., Sagebiel D. (2018). Fatal Neurotoxicosis in Dogs Associated with Tychoplanktic, Anatoxin-a Producing Tychonema sp. in Mesotrophic Lake Tegel, Berlin. Toxins.

