# Supplementary material for: Multi-Toxin Occurrences in Ten French Water Resource Reservoirs
Source: Toxins (Basel). 2018 Jul 9;10(7):283. doi: 10.3390/toxins10070283 (PMC6071237; doi:10.3390/toxins10070283)
Supplement: Supplementary file 1 [file toxins-10-00283-s001.pdf]

Supplementary Materials: Multi-Toxin Occurrences in Ten French Water Resource Reservoirs

Frederic Pitois, Jutta Fastner, Christelle Pagotto and Magali Dechesne

Table S1. Observed cyanobacterial taxa with known or suspected (in parenthesis) potential toxin production (detailed references are provided below).

Order	Species	MCs	ATX	PSP	CYN
Nostocales	*Anabaena aphanizomenoïdes*	+ [1]			
	Anabaena flos-aquae	+ [2]	+ [3]		
	Anabaena planctonica	+ [4]	+ [5]		
	Anabaena spiroides		+ [5]		
	Anabaenopsis arnoldii	+ [6]			
	Aphanizomenon flos-aquae		+ [7]	+ [8]	+ [9]
	Aphanizomenon gracile			+ [10]	+ [11]
	Aphanizomenon issatschenkoï		+ [12]	+ [13]	
	Cylindrospermopsis raciborskii			+ [14]	+ [15]
	Raphidiopsis brookii			+ [16]	
	Raphidiopsis curvata				+ [17]
Chroococcales	*Microcystis aeruginosa*	+ [18]	+ [5]	+ [19]	
	Microcystis botrys	+ [20]			
	Microcystis flos-aquae	+ [21]			
	Microcystis ichtyoblabe	+ [22]			
	Microcystis viridis	+ [18]			
	Microcystis weisenbergii	+ [23]			
	Woronichinia naegeliana	+ [24]			
Oscillatoriales	*Limnothrix redekeii*	+ [25]			
	Phormidium splendidum	+ [26]			
	Planktothrix agardhii	+ [27]			
	Planktothrix clathrata	+ [20]			
	Planktothrix rubescens	+ [28]	+ [29]		
	Pseudanabaena limnetica	+ [30]	+ [7]		
	Pseudanabena mucicola	+ [23]			

Reference

1. Sabour, B.; Loudiki, M.; Oudra, B.; Vasconcelos, V.; Oubraim, S.; Fawzi, B. Dynamics and toxicity of *Anabaena aphanizomenoides* (Cyanobacteria) waterblooms in the shallow brackish Oued Mellah lake (Marocco). *Aquat. Ecosyst. Health Manage.* **2005**, *8*, 1–10.
2. Sivonen, K.; Skulberg, O.M.; Namikoshi, M.; Evans, W.R.; Carmichael, W.W.; Rinehart, K.L. Two methyl ester derivatives of microcystins, cyclic heptapeptide hepatotoxins, isolated from *Anabaena flos-aquae* strain cya 83/1. *Toxicon* **1992.**, *30*, 1465–1471.
3. Sivonen, K.; Himberg, K.; Luukkainen, R.; Niemelä, S.I.; Poon, G.K.; Codd, G.A. Preliminary characterization of neurotoxic cyanobacteria blooms and strains from Finland. *Toxicity Assess.* **1989**, *4*, 339–352.
4. Sant'Anna, C.L.; Azevedo, M.T.P.; Werner, V.R.; Dogo, C.R.; Rios, F.R.; de Carvalho, L.R. Review of toxic species of cyanobacteria in Brazil. *Algological studies* **2008**, *126*, 251–265.
5. Park, H.-D.; Watanabe, M.F.; Harada, K.-I.; Nagai, H.; Suzuki, M.; Watanabe, M.; Hayashi, H. Hepatotoxin (microcystin) and neurotoxin (anatoxin-a) contained in natural blooms and strains of cyanobacteria from Japanese freshwaters. *Nat. Toxins* **1993**, *1*, 353–360.
6. Mohamed, Z.A.; Al Shehri, A.M. Microcystin-producing blooms of *Anabaenopsis arnoldi* in a potable mountain lake in Saudi Arabia. *FEMS Microbiol. Ecol.* **2009**, *69*, 98–105.

7. Osswald, J.; Rellán, S.; Gago-Martinez, A.; Vasconcelos, V. Production of Anatoxin-a by cyanobacterial strains isolated from Portuguese fresh water systems. *Ecotoxicol.* **2009**, *18*, 1110–1115.
8. Liu, Y.; Chen, W.; Li, D.; Shen, Y.; Li, G.; Liu, Y; First report of aphantoxins in China-waterblooms of toxigenic *Aphanizomenon flos-aquae* in lake Dianchi. *Ecotoxicol. Environ. Saf.* **2006**, *65*, 84–92.
9. Preußel, K.; Stüken, A.; Wiedner, C.; Chorus, I.; Fastner, J. First report on cylindrospermopsin producing *Aphanizomenon flos-aquae* (Cyanobacteria) isolated from two German lakes. *Toxicon* **2006**, *47*, 156–162.
10. Ballot, A.; Fastner J.; Wiedner, C. Paralytic shellfish poisoning toxin-producing cyanobacterium *Aphanizomenon gracile* in Northeast Germany. *Appl. Environ. Microbiol.* **2010**, *76*, 1173–1180.
11. Kokocinski, M.; Mankiewicz-Boczek, J.; Jurczak, T.; Spoof, L.; Meriluoto, J.; Rejmonczyk, E.; Hautala, H.; Vehniainen, M.; Pawelczyk, J.; Soininen, J. *Aphanizomenon gracile* (Nostocales), a cylindrospermopsin-producing cyanobacterium in Polish lakes. *Environ. Sci. Pollut. Res. Int.***2013**, *20*, 5243-5264.
12. Wood, S.A.; Rasmussen, J.P.; Holland, P.T.; Campbell, R.; Crow, A.L.M. First report of the cyanotoxin anatoxin-a from *Aphanizomenon issatschenkoï* (Cyanobacteria). *J. Phycol.* **2007**, *43*, 356–365.
13. Pereira, P.; Onodera, H.; Andrinolo, D.; Franca, S.; Araújo, F.; Lagos, N.; Oshima, Y. Paralytic Shellfish toxins in the freshwater cyanobacterium *Aphanizomenon flos-aquae*, isolated from Montargil reservoir, Portugal. *Toxicon* **2000**, *38*, 1689–1702.
14. Lagos, N.; Onodera, H.; Zagatto, P.A.; Andrinolo, D.; Azevedo, S.M.F.Q.; Oshima, Y. The first evidence of paralytic shellfish toxins in the freshwater cyanobacterium *Cylindrospermopsis raciborskii*, isolated from Brazil. *Toxicon* **1999**, *37*, 1359–1373.
15. Hawkins, P.R.; Runnegar, M.T.C.; Jackson, A.R.B.; Falconer, I.R. Severe hepatotoxicity caused by the tropical cyanobacterium (blue-green alga) *Cylindrospermopsis raciborskii* (Woloszynska) Seenaya and Subba Raju isolated from a domestic water supply reservoir. *Appl. Environ. Microbiol.* **1985**, *50*, 1292–1295.
16. Soto-Liebe, K.; Méndez, M.A.; Fuenzalida, L.; Krock, B.; Cembella, A.; Vásquez, M. PSP toxin release from the cyanobacterium *Raphidiopsis brookii* D9 (Nostocales) can be induced by sodium and potassium ions. *Toxicon* **2012**, *60*, 1324–1334.
17. Li, R.; Carmichael, W.W.; Brittain, S.; Eaglesham, G.K.; Shaw, G.R.; Liu, Y.; Watanabe, M.M. First report of the cyanotoxins cylindrospermopsin and deoxycylindrospermopsin from *Raphidiopsis curvata* (Cyanobacteria). *J. Phycol.* **2002**, *37*, 1121–1126.
18. Shirai, M.; Ohtake, A.; Sano, T.; Matsumoto, S.; Sakamoto, T.; Sato, A.; Aida, T.; Harada, K.-I.; Shimada, T.; Suzuki, M.; Nakano, M. Toxicity and toxins of natural blooms and isolated strains of Microcystis spp. (Cyanobacteria) and improved procedure for purification of cultures. *Appl. Environ. Microbiol.* **1991**, *57*, 1241–1245.
19. Sant'Anna, C. L.; de Carvalho, L.R.; Fiore, M.F.; Silva-Stenico, M. E.; Lorenzi, A.S.; Rios, F.R.; Konno, K.; Garcia, C.; Lagos, N. Highly toxic *Microcystis aeruginosa* strain, isolated from Sao Paulo-Brazil, produce hepatotoxins and paralytic shellfish poison neurotoxins. *Neurotoxicity Res.* **2011**, *19*, 389–402.
20. Henriksen P. Toxic cyanobacteria/blue-green algae in Danish fresh waters. Ph.D. Thesis, Dept. of Phycology, Botanical Institute, University of Copenhagen, Denmark, **1996**.
21. Gkelis, S.; Lanaras, T.; Sivonen, K. Cyanobacterial Toxic and Bioactive Peptides in Freshwater Bodies of Greece: Concentrations, Occurrence Patterns, and Implications for Human Health. *Mar. Drugs* **2015**, *13*, 6319-6335.
22. Nguyen, V.L.A.; Tanabe, Y.; Matsuura, H.; Kaya, K.; Watanabe, M.M. Morphological, biochemical and phylogenetic assessments of water-bloom-forming tropical morphospecies of Microcystis (Chroococcales, cyanobacteria). *Phycological Res.* **2012**, *60*, 208–222.
23. Oudra, B.; Loudiki, M.; Vasconcelos, V.; Sabour, B.; Sbiyyaa, B.; Oufdou, Kh.; Mezrioui, N. Detection and quantification of microcystins from cyanobacteria strains isolated from reservoirs and ponds in Marocco. *Environ. Toxicol.* **2002**, *17*, 32–39.
24. Oberholster, P.J.; Botha, A.-M.; Cloete, T.E. Toxic cyanobacterial blooms in a shallow, artificially mixed urban lake in Colorado, USA. *Lakes Reservoirs Res. Manage.* **2006**, *11*, 111–123.
25. Pineda-Mendoza, R.M.; Olvera-Ramirez, R.; Martinez-Jeronimo, F. Microcystins produced by filamentous cyanobacteria in urban lakes. A case study in Mexico City. *Hidrobiologica* **2012**, *22*, 290–298.
26. Aboal, M.; Puig, M.A.; Asencio, A.D. Production of microcystins in calcareous Mediterranean streams: The Alharabe River, Segura River basin in south-east Spain. *J. Appl. Phycol.* **2015**, *17*, 231–243.

27. Luukkainen, R.; Sivonen, K.; Namikoshi, M.; Färdig, M.; Rinehart, K.L.; Niemelä, S. Isolation and identification of eight microcystins from thirteen *Oscillatoria agardhii* strains and structure of a new microcystin. *Appl. Environ. Microbiol.* **1993**, *59*, 2204–2209.
28. Briand, J.-F.; Jacquet, S.; Flinois, C.; Avois-Jacquet, C.; Maisonnette, C.; Leberre, B.; Humbert J.-F. Variations in the microcystin production of *Planktothrix rubescens* (Cyanobacteria) assessed from a four-year survey of lac du Bourget (France) and from laboratory experiments. *Microb. Ecol.* **2005**, *50*, 418–428.
29. Viaggiu, E.; Melchiorre, S.; Volpi, F.; Di Corcia, A.; Mancini, R.; Garibaldi, L.; Crichigno, G.; Bruno, M. Anatoxin-a toxin in the cyanobacterium *Planktothrix rubescens* from a fishing pond in northern Italy. *Environ. Toxicol.* **2004**, *19*, 191-197.
30. Maršálek, B.; Bláha, L.; Babica, P. Analyses of microcystins in the biomass of *Pseudanabaena limnetica* collected in Znojmo reservoir. *Czech Phycol.* **2003**, *3*, 195–197.
